# Nutritional Supplements and Neuroprotective Diets and Their Potential Clinical Significance in Post-Stroke Rehabilitation

**DOI:** 10.3390/nu13082704

**Published:** 2021-08-05

**Authors:** Ewa Zielińska-Nowak, Natalia Cichon, Joanna Saluk-Bijak, Michał Bijak, Elzbieta Miller

**Affiliations:** 1 Department of Neurological Rehabilitation, Medical University of Lodz, Milionowa 14, 93-113 Lodz, Poland; ewa.zielinska@umed.lodz.pl; 2 Biohazard Prevention Center, Faculty of Biology and Environmental Protection, University of Lodz, Pomorska 141/143, 90-236 Lodz, Poland; natalia.cichon@biol.uni.lodz.pl (N.C.); michal.bijak@biol.uni.lodz.pl (M.B.); 3 Department of General Biochemistry, Faculty of Biology and Environmental Protection, University of Lodz, Pomorska 141/143, 90-236 Lodz, Poland; joanna.saluk@biol.uni.lodz.pl

**Keywords:** nutritional supplements, malnutrition, neuroprotective diets, stroke, rehabilitation

## Abstract

Nutrition and rehabilitation are crucial in post-stroke recovery, especially in the elderly. Since stroke is the leading cause of long-term disability, there is a need to promote special, individually tailored nutrition strategies targeting older patients with low motor ability. Chronic stroke survivors have higher risk of developing nutrition-related chronic diseases, such as sarcopenia, anemia, type 2 diabetes mellitus and osteoporosis. Moreover, reduced motor activity, cognitive impairment and depression might be aggravated by poor malnutrition status. Accumulated data suggest that nutritional supplements and neuroprotective diets can be associated with better effectiveness of post-stroke rehabilitation as well as brain recovery. Therefore, this review focuses on preventive strategies that can improve dietary intake and change dietary patterns. We highlight the importance of neuroprotective diets, the problem of dysphagia and the role of nutrition in rehabilitation. This article focuses on potential nutritional supplements and neuroprotective diets that may have an impact on functional recovery during and after rehabilitation. Moreover, a new approach to post-stroke neuroplasticity including the use of agents from marine sources such as fucoxanthin and tramiprosate as compounds that might be used as potential neuroprotectants with antioxidative and anti-inflammatory properties is introduced.

## 1. Introduction

Stroke is one of the most common causes of disability in adults. Currently, despite introducing thrombolysis and thrombectomy treatment, there is still high demand to prevent neuronal death and neurological dysfunction in post-stroke patients. Moreover, patients after stroke have about a 43% higher risk of stroke reoccurrence over 10 years with an increased annual rate of 4% [1].

Therefore, long-term management of risk factors, including proper nutrition, plays a major role in medical care, especially during the rehabilitation process. Prevention of stroke recurrence is mainly based on changes in behavioral strategies and lifestyle factors. The main lifestyle modifiable factors are smoking, diet, obesity, alcohol and physical activity [2].

Generally, effective rehabilitation requires a holistic approach. In most cases, post-stroke rehabilitation begins the day after stroke, but it is a very short program due to the diagnostic and therapeutic process. The intensity of rehabilitation is tailored to patients’ individual needs. However, during the first 2–3 months, when neuroplasticity is very active, the level of patients’ activity is gradually accelerated and focused on functional, cognitive and emotional improvement [3].

Most patients are able to tolerate an increased duration of rehabilitation programs, especially in specialized post-stroke rehabilitation services or units. In different countries, there are different recommendations for the duration of the rehabilitation program [4].

In Poland, the minimum duration of the whole program including physiotherapy, cognition and speech therapy is 150 min daily with a one-day break on Sunday.

One of the most important factors determining post-stroke rehabilitation effectiveness is appropriate nutrition, adjusted to patient’s medical history and increased demands during the complex biochemical processes of brain recovery and high physical and cognitive activity. Moreover, malnutrition is associated with poor clinical and functional outcomes in post-stroke patients, with greater incidence of infections and pressure sores as well as higher lengths of hospitalizations [5]. There are huge differences between reports in the prevalence of malnutrition in patients after a stroke, ranging from 6.1 to even 62%, whilst other studies have reported the rate ranges from 8 to 49%. Routine tests of nutritional status are recommended for all post-stroke patients in order to enhance their potential for recovery and prevent the development of malnutrition during rehabilitation [6,7].

**Figure 1:**
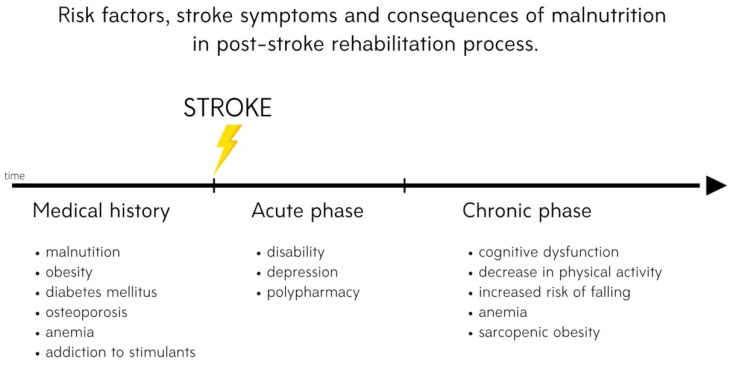
Risk factors, stroke symptoms and consequences of malnutrition in the post-stroke rehabilitation process.

The aim of this review is to present risk factors for the development of malnutrition and nutrition-related chronic diseases in post-stroke patients (Figure 1); the role of nutrition; and the most effective strategies to prevent malnutrition, including neuroprotective diets and nutritional supplements, in post-stroke rehabilitation. This article focuses on evidence that nutritional status has a correlation with functional recovery during and after rehabilitation. Moreover, a new approach to post-stroke neuroplasticity including treatment with agents from marine sources such as fucoxanthin and tramiprosate as compounds that might be used as potential neuroprotectants with antioxidative and anti-inflammatory properties is introduced.

Currently, there is great interest in dietary supplementation to support rehabilitation, alleviate vascular diseases, decrease ischemic brain damage and enhance processes of spontaneous recovery and neuroplasticity. Data from several clinical studies show medical benefits from dietary supplementation not only in functional but also in cognitive and emotional status in post-stroke patients.

## 2. Risk of Malnutrition after Stroke

Malnutrition is common in patients with neurological conditions, including stroke. The causes of this abnormality are directly related to neurological diseases, such as cognitive functions and disorder of consciousness, neurogenic vomiting, neurogenic dysphagia, depression, motor deficit and gastrointestinal dysfunction [8,9,10].

A significant element of therapeutic and preventive management in central nervous system (CNS) disorders is proper assessment and monitoring of the nutritional status. The reasons for the reduced consumption of nutrients include dysphagia, obstruction or stricture of the esophagus, disability, a general deterioration in condition, increased catabolic processes, digestive disorders and pharmacotherapy [11].

Malnutrition is a well-documented negative prognostic factor both in the general population and in various patient groups. It greatly increases the risk of complications such as pressure ulcers and infections (especially of the respiratory system), electrolyte disturbances, coagulation disorders, anemia, osteoporosis and bradycardia and also reduces the quality of life. Furthermore, it influences the hospitalization time and the number of stays in intensive care units and reduces the effectiveness of rehabilitation [10]. Besides, malnutrition also increases mortality from roughly 30 to 180 days after the ischemic incident. In the Feed Or Ordinary Diet (FOOD) clinical trial it was noted that the odds ratio (OR) of death in the group of post-stroke patients with malnutrition was 2.32 (95% confidence interval (CI) 1.78–3.02) compared to post-stroke patients with normal nutritional status [12].

The most important neurological cause of nutritional disorders is dysphagia, which includes disturbances in the swallowing process at any of its stages: from taking food into the mouth, through keeping it in the buccal cavity, chewing and shaping it, to transporting it from the oral cavity through the throat and esophagus to the stomach. The prevalence of dysphagia in the general population is estimated at 7%; this percentage increases with age, and in the elderly, it reaches 50% [13,14,15]. In patients with CNS disease, the most common cause of dysphagia, the prevalence of dysphagia is estimated to be 50% [16,17].

Neurogenic dysphagia can be caused by disturbances in the coordination of individual swallowing phases, paresis of the muscles involved in the act of swallowing, abnormal muscle tone, disturbance in swallowing–breathing coordination, disturbance of sensation in the mouth or throat, involuntary movements, disturbances in the central control of swallowing or most commonly a combination of these symptoms [18,19,20]. Neurogenic dysphagia is most often associated with neurological deficits that make it difficult to adopt and maintain a proper position while eating and can significantly reduce the critical insight into the symptoms presented. Patients with swallowing disorders also often have sensory disorders, undergo pharmacotherapy that can reduce muscle tone and attention and have problems with dentition. Importantly, a major impediment in the diagnosis of dysphagia is the frequent lack of patients’ consciousness of the problem, which indicates the need for routine assessment of possible swallowing disorders by medical and nursing staff [14,15,16,19].

The most important potentially fatal consequences of dysphagia, regardless of etiology, include dehydration, malnutrition and aspiration to the respiratory system, leading to Mendelson’s syndrome [21,22]. For this reason, early diagnosis of dysphagia and proper nutritional intervention are of key importance for the prognosis of patients with CNS diseases. In all patients with dysphagia, it is extremely important to assess and monitor the nutritional status, including qualitative and quantitative evaluation of consumed food and fluid. This is extremely important because it enables the early identification of patients requiring nutritional support or treatment. In addition, careful oral hygiene, the use of anti-reflux procedures and safe feeding reduce the risk of pneumonia. Hospitalized patients requiring diet modification should have access to properly modified food and fluids [23].

## 3. Nutrition-Related Chronic Diseases in Post-Stroke Patients

### 3.1. Osteoporosis

Loss of bone mineral density (BMD) is a common symptom in post-stroke patients. Bone loss begins immediately after stroke and progresses for the first few months. Then, it stabilizes at a lower rate but still does not recede for at least a year [24]. The pathology of post-stroke osteoporosis is complex, as it may involve reduced bone load, reduced mobility, paresis and changes in muscle mass and strength. However, it may also be related to nutritional deficits [24,25]. Long-term hypocalcemia might also contribute to osteoporotic problems. In a recent study by Siotto et al. on calcium levels in post-stroke patients, it was discovered that 26.7% of patients had total calcium levels below the reference range, and it was suggested that serum calcium levels might be correlated with patients’ outcomes during the rehabilitation program. What is more, total protein and albumin were below the reference range in about 77% and 67% of patients. Authors recommend measurements of calcium levels at the beginning and during the rehabilitation program in order to apply appropriate supplementation or dietary program [26]. Osteoporosis not only negatively influences functional recovery, but also is associated with poor cognitive function during the acute and recovery stroke phases [27]. In addition to medications, treatment should also include muscle-strengthening training; resistance training; and appropriate supplementation, which includes vitamin D, folic acid, mecobalamin and calcium [24,25]. It is worth mentioning that only 15.5% of stroke patients receive treatment to prevent bone loss and that pharmacological treatment often prescribed after stroke, such as statins or warfarin, might have a negative influence on bone density [28].

### 3.2. Anemia

Anemia might be considered as one of the stroke risk factors. In post-stroke patients, it is a predictor of poor outcomes with increased morbidity and mortality [29,30]. Patients with this condition are more likely to have recurrent stroke [31]. In case of anemia, administration of iron is recommended, although excessive iron supplementation might cause side effects. If oral administration of iron is insufficient, it is worth considering the newer intravenous iron complexes (i.e., iron sucrose complexes), which are safer and easily administered [29,32]. In post-stroke patients, anemia should be treated as soon as possible due to the fact that hemoglobin improvement has a positive influence on functional recovery and may reduce the duration of hospitalization [33,34]. When comparing FIM scores between post-stroke patients with and without anemia, the nonanemic group had a significantly higher FIM score improvement and FIM efficiency [33].

### 3.3. Sarcopenia

Sarcopenia is also a frequent occurrence after stroke with an increased incidence of 14 to 54% [35]. Risk factors for sarcopenia include older age, lack of physical activity and malnutrition, which largely affect patients after stroke. Changes in muscle tissue begin within hours after stroke, and reduction in muscle mass is rapid, which can lead to physical function reduction or even disability [36]. Several factors contribute to sarcopenia occurrence; therefore, it requires an interdisciplinary approach, combining proper rehabilitation and nutritional support. The rehabilitation program should primarily consist of resistance training, but also walking and ADL training, paralyzed limb facilitation and aerobic training to prevent sarcopenic obesity [37]. When it comes to nutrition, studies suggest increased protein intake, high-energy and high-protein meals and leucine-enriched amino acid supplementation [37,38,39].

### 3.4. Diabetes Mellitus

Diabetes mellitus (DM) is another factor predicting poor outcome with higher mortality after stroke [40,41,42]. In an interesting study by Zhang Y. et al. it was suggested that patients with DM are more likely to suffer from late-onset post-stroke depression [43]. The prevalence of DM is significant; it has been determined that one-third of post-stroke patients have this disease [42]. Both the prevention of stroke among DM patients and post-stroke treatment should primarily include normalizing blood glucose levels [41]. Lifestyle changes such as controlling body weight, minimizing total fat intake (especially saturated fat), consuming a low-carbohydrate diet, consuming low sodium and cholesterol and augmenting fiber intake may also be beneficial [41,44].

## 4. Preventive Strategies

The serious socioeconomic problem of stroke requires the search for new neuroprotective management, both to reduce the number of new cases and to reduce the size of the stroke infarct and its consequences. The provision of neuroprotective compounds is therefore essential not only in the primary prevention of stroke, but also in post-stroke recovery.

### Neuroprotective Diets

Currently, relationships between nutritional status, diet and the functioning of the central nervous system are increasingly recognized. The analysis of the literature shows the effect of individual nutrients from food on the nervous system, while multiannual population studies on eating behaviors and habits indicate the protective effect of the diet. It has been shown that certain types of diets have an effect on life expectancy, mortality, risk of cardiovascular disease and cognitive impairment. Diet as a potentially modifiable lifestyle element has a potential for primary and secondary prevention of risk factors for various diseases, including stroke. Both qualitative (minerals, vitamins, fats and proteins) and quantitative (number of calories consumed) aspects of the diet are considered.

Taking into account the relationship between the consumed food and the health condition, it was noticed that individual groups of products or individual behaviors affect not so much health, but the overall model of nutrition. The Mediterranean diet (MD) is one such model, characterized by food diversity; low calories; high consumption of fruit and vegetables, legumes, nuts and seeds, grains and fish; and relatively low consumption of meat and dairy. A meta-analysis by Papadaki et al. found that regular use of the MD reduced the risk of stroke (relative risk (RR) = 0.67, 95% confidence interval (CI) 0.35–0.98; I2 = 0%) [45], and a meta-analysis, published in the Cochrane Library, assessing both the effect of the MD and the quality of clinical trials showed that the MD reduced the number of strokes in primary prevention (hazard risk (HR) 0.60, 95% CI 0.45–0.80; moderate-quality evidence). Low-quality evidence was also observed for a reduction in death and overall mortality from cardiovascular disease in secondary prevention (HR 1.0, 95% CI 0.81–1.24; HR 0.81, 95% CI 0.50–1.32, respectively). Moreover, the impact of the MD on the improvement of risk factors in primary prevention was demonstrated: reduction in triglycerides (−0.09 mmol/L, 95% CI −0.16 to 0.01), LDL cholesterol (−0.15 mmol/L, 95% CI −0.27 to −0.02; moderate-quality evidence) and diastolic (−2.0 mmHg, 95% CI −2.29 to −1.71) and systolic blood pressure (−2.99 mmHg, 95% CI −3.45 to −2.53) [46]. The beneficial effect of the MD is probably related to synergistic interactions between the various ingredients as a whole of the nutritional regimen [47]. A potential mechanism underlying the effect of the MD on the vascular system may be associated with a beneficial impact on endothelium-dependent vasoreactivity and insulin resistance [48,49,50], as well as with antioxidant and anti-inflammatory activity [51,52,53].

The Mediterranean–DASH Intervention for Neurodegenerative Delay (MIND) diet is a hybrid of the Mediterranean diet and the Dietary Approaches to Stop Hypertension (DASH) diet, most often used in hypertension cardio- and cerebrovascular diseases. In 2015, Morris et al. presented a nutritional model that aimed to improve the functioning of the brain and nervous system, in particular cognitive functions, as well as reduce the risk of developing and progressing neurodegenerative diseases [54]. Based on large cohort studies, they found that consumption of at least six portions per week of green leafy vegetables (lettuce, cabbage, kale, and spinach), as well as berry fruits (strawberries, blueberries), significantly improved cognitive function. The effectiveness of the MIND diet has been confirmed in several cohort studies, which showed improvement of cognitive functions, including episodic, working and semantic memories; perceptual speed; and visuospatial ability [54,55,56]. In 2019, Cherian et al. conducted a study in which they assessed the effectiveness of the MIND diet in preventing post-stroke cognitive decline. One hundred six patients were enrolled; they were followed on average for 5.9 years. Cognitive functions were measured in five domains (semantic, episodic and working memories; perceptual orientation; and speed) using annual structured clinical estimation. They showed that after adjusting for sex, age, education, late-life cognitive activity, APOE-ε4, caloric intake, smoking and physical activity, the highest and lowest terciles of MIND diets had a lower rate of global cognitive decline (β 0.08, 95% CI 0.1–0.15) and lower decline in semantic memory (β 0.07, 95% CI: 0.00, 0.14) and perceptual speed (β 0.07, 95% CI: 0.00, 0.14) [57].

The prohealth properties of olive oil are associated with not only its content of over 30 types of phenolic compounds, but also the presence of MUFAs, mainly oleic acid and omega-6 and omega-3 PUFAs. The effect of olive oil consumption in primary prevention of stroke is assessed by measuring the level of oleic acid in the plasma. The Three-City Study found that consuming a lot of olive oil, compared with minimal or no consumption, decreased the risk of stroke by approximately 73% (95% CI 10−92%, *p* = 0.03), and the olive-oil-consuming subjects had lower plasma triglyceride concentration and body mass index (BMI) [58]. Similarly, meta-analyses evaluating the relationship between olive oil consumption in primary prevention suggested a neuroprotective effect in relation to vascular diseases [59,60]. Moreover, studies in animal models showed that olive oil reduced neuronal apoptosis, infarct volume, blood–brain barrier (BBB) permeability and brain edema and improved neurological deficits in rodents with I/R injury [61,62,63].

One variation of neuroprotective diet is the ketogenic diet (KD), which is of increasing interest with respect to various diseases of the nervous system. The main assumption of KD is the use of fatty acids as the main source of cellular energy; therefore, it is characterized by a high fat content (about 80–90%), the maximum elimination of carbohydrates and less than 30% protein. KD causes an increased production of ketone bodies: acetoacetate (AcC), β-hydroxybutyrate (BHB) and acetone, alternative sources of cellular energy to glucose [64]. KD is widely used in the nonpharmacological treatment of epilepsy. The antiseizure effect of KD on the molecular level may be associated with ATP-sensitive potassium (K_ATP_) channels, BCL-2-associated agonists of cell death, vesicular glutamate transporters, lactate dehydrogenase and adenosine A_1_ receptors (A_1_Rs) [65]. However, in recent years, it has been suggested that the use of KD may have therapeutic potential in the treatment of neurodegenerative diseases, ischemia and traumatic brain injury. The mechanism of stroke-induced nerve tissue injury is mainly related to excitotoxicity, intracellular calcium load, production of reactive oxygen species, inflammation, apoptosis and mitochondrial dysfunction. Thus, the pathomechanism of I/R injury is to some extent common in neurodegenerative diseases. Currently, only studies in animal models showing the neuroprotective effects of ketones after stroke are available. They exhibit neurorestorative activity by reducing markers of apoptosis, inhibiting caspase 3, as well as reactive oxygen species production [66]. Maalouf et al. observed that inhibition of mitochondrial reactive oxygen species (ROS) generation by ketones was associated with increased nicotinamide adenine dinucleotide (NADH) oxidation in the electron transport chain [67]. The antioxidant effect of ketones is related to the improvement of mitochondrial antioxidant capacity with a simultaneous increase in the level of glutathione [68]. Furthermore, Julio-Amilpas et al. confirmed that BHB has a protective effect on cortical neurons by inhibiting the production of ROS and increasing the production of adenosine triphosphate (ATP) [69]. Moreover, studies conducted by Suzuki et al. showed that BHB reduced brain edema and prolonged survival time by improving energy metabolism in an animal model [70], as well as reducing stroke area and neurological deficits in animals [71]. In turn, Yang et al. found that KD caused an increase in regional blood flow in the brain, a decrease in the volume of a stroke and an enhancement in the extracellular level of adenosine. The proposed mechanism is related to the activation of adenosine A1 receptor (A1R), causing phosphorylation of protein kinase B (Akt) and extracellular signal-regulated kinase 1/2 (ERK1/2), and to the upregulation of hypoxia-inducible factors (HIFs) and HIF-regulated genes (EPO and VEGF) [72]. Moreover, Shaafi et al. suggested that KD improved early behavioral and motor outcomes in rats with induced stroke [73].

## 5. The Role of Nutritional Status in Post-Stroke Rehabilitation

Rehabilitation plays a crucial role in post-stroke recovery. It not only can affect physical functioning, but also has a positive effect on psychosocial well-being. A proper exercise program can positively influence patients’ mobility, balance, gait, muscle strength, pain, cognition, memory and, in general, quality of life [74]. Taking this into consideration, post-stroke care requires a multidisciplinary approach, including adequate nutrition, in order to provide patients with ideal conditions for rehabilitation.

There is some evidence that nutritional status is correlated with functional recovery during and after rehabilitation. The elderly, including those after stroke, often experience malnutrition, nutritional deficits or dehydration which also have a negative impact on their physical functioning, strength and independence in activities of daily living (ADL) [75]. Patients with higher Geriatric Nutritional Risk Index (GNRI) at admission have lower improvements in Functional Independence Measure (FIM), which suggests that GNRI may predict FIM gain during hospitalization [76].

In a study on 178 post-stroke patients with malnutrition, there was an association between improvement in nutritional status and ADL. Measurements were performed at the beginning and at the end of the rehabilitation program and were taken with FIM. The factor that was most likely related to the improvements in ADL was the maintenance of body weight. This study has also shown that patients who at the end of the rehabilitation program were still malnourished also had worse rehabilitation results in comparison to patients with a greater nutritional improvement during rehabilitation [77].

A recent study conducted in Japan assessed the impact of frequent individualized diet support during a rehabilitation program on patient outcomes. Four hundred fifty-four patients underwent rehabilitation treatment, consisting of paralyzed limb facilitation, range-of-motion exercises, basic movement training, walking training, resistance training and aerobic exercises using an ergometer, aimed at improving endurance and weight loss to combat obesity, ADL training and dysphagia rehabilitation. Nutritional management was preceded by a thorough nutritional screening and assessment, and then provision of high-energy and high-protein meals for malnourished patients, calorie restriction for weight reduction and provision of adequate protein for maintaining muscle mass in obese patients were applied. Rehabilitation outcomes were measured with FIM, and it was found that frequent individualized diet support resulted in better functional recovery [78]. There has also been an interesting study in which the authors investigated the relationship between BMI and functional recovery after stroke in rehabilitation wards. It has shown that patients with obesity (≥27.5 kg/m^2^) had significantly better results than other groups (underweight, standard, overweight) in FIM gain [79]. Similar results were obtained in a study on 819 patients in a post-stroke rehabilitation hospital. However, in that case, the overweight group had the highest FIM efficiency (FIM score changes per day), and the underweight group had the lowest FIM efficiency [80]. In Table 1 we summarize presented data concerning the correlation between nutritional status and functional recovery after stroke (Table 1).

## 6. Impact of Nutritional Supplementation on Post-Stroke Rehabilitation

Nutritional supplements are recommended in patients who are undergoing rehabilitation very often due to the higher demand for bioactive compounds during complex processes of recovery.

In a randomized, prospective, double-blind, single-center study on 116 post-stroke, undernourished patients during rehabilitation, a group with intensive supplementation achieved better results than the group with standard supplementation. The outcomes that improved more included total FIM score, FIM motor subscore and 2 min and 6 min timed walk tests [81].

### 6.1. Amino Acid Supplementation

Amino acid supplementation seems to be a very important factor in the prevention of muscle protein hypercatabolism that often occurs after stroke. Rehabilitation in combination with amino acid supplementation can contribute to the improvement of muscle mass and functional efficiency in the early post-stroke phase [82]. Patients without essential amino acid supplementation continued to lose muscle mass even 76 days after stroke [83].

In order to increase muscle protein synthesis and reduce muscle soreness, post-exercise consumption of leucine-enriched amino acids is recommended because leucine plays a crucial role in triggering postprandial muscle protein synthesis through the mammalian target of rapamycin (mTOR) pathway [38]. Specific supplementation with leucine-enriched amino acids has proven to be effective in post-stroke patients with sarcopenia. In a randomized controlled trial, patients with sarcopenia were divided into two groups, both of which performed low-intensity resistance training during a rehabilitation program (8 weeks), but only the intervention group was supplemented with leucine-enriched amino acids (3 g of leucine 40% enriched essential amino acids and 9.7 g of carbohydrate). The improvement in FIM score was present in both groups, as was the improvement in skeletal muscle mass index (SMI) and handgrip strength, although the improvement in the intervention group was significantly greater than that in the control group [38]. Ikeda et al. investigated whether the timing of intake of supplemental branched-chain amino acids (BCAAs) had an effect on body composition and physical functioning. Supplements contained 3.5 g of amino acids, 6.5 g of protein and 40 IU of vitamin D per 125 mL. Patients were divided into two groups, depending on the timing of supplement intake: breakfast and post-exercise. Both groups underwent a rehabilitation program for 2 months, with two sessions daily. Muscle mass results were similar in both groups; however, BCAA intake with breakfast was more effective for physical performance and decreasing body fat mass [84].

### 6.2. Vitamin D Supplementation

Vitamin D deficiency is a common problem among stroke survivors, and it is associated with decreased muscle strength, balance and physical performance [85,86]. Although the results of studies on the effect of vitamin D supplementation on post-stroke rehabilitation are contradictory, most of them indicate its potential to support functions of the nervous system and enhance the rehabilitation process. Siotto et al. have drawn attention to an interesting issue: not only does serum concentration of vitamin D may affect post-stroke recovery during rehabilitation, but also single nucleotide polymorphisms and promoter methylation in genes that affect circulating vitamin levels may influence patients’ outcomes. According to the authors, it is crucial to measure levels of 25(OH)D and determine the presence or absence of CYP2R1, CYP27B1, CYP4A1 and VDR polymorphisms and analyze their methylation before and after the rehabilitation program [87].

Most recent evidence suggests that vitamin D supplementation may positively affect lower extremity motor functioning and ambulation. Utkan Karasu et al. divided 76 patients into two groups, where the treatment group received 50,000 IU of vitamin D weekly for 4–12 weeks and the control group did not receive any vitamin D supplementation during rehabilitation. After rehabilitation, program patients receiving vitamin D had higher changes in scores of Brunnstrom recovery staging (lower extremity) and functional ambulation classification (FAC) (*p* = 0.005 and *p* = 0.018) [88]. This is consistent with what has been previously found by Gupta et al., who found that post-stroke patients supplemented with vitamin D (600,000 IU single intramuscular injection and 60,000 IU once a month) plus elemental calcium (one gram per day) for 6 months had better results in modified Rankin scale than the control group receiving only usual care [86]. The effect of vitamin D supplementation (300,000 IU injection vs. saline injection in control group) on rehabilitation outcomes and balance in hemiplegic post-stroke patients has also been investigated. Patients were tested with Brunnstrom recovery staging (BRS), functional ambulation classification (FAC), modified Barthel index and Berg balance scale (BBS) at the beginning and at the end of the three-month rehabilitation program, and the outcomes of the BBS and modified Barthel index were significantly different between the two groups. Improvements in the vitamin D supplementation group were higher; however, BRS and FAC results were not [85]. There is some evidence that vitamin D supplementation may not improve the rehabilitation process after stroke, although it might be related to the dose of vitamin D, which in the study of Momosaki et al. was smaller (2000 IU per day) than in previously cited results [89].

The presented research results regarding the influence of supplementation on functional recovery during post-stroke rehabilitation are summarized in Table 2 (Table 2).

**Figure 2:**
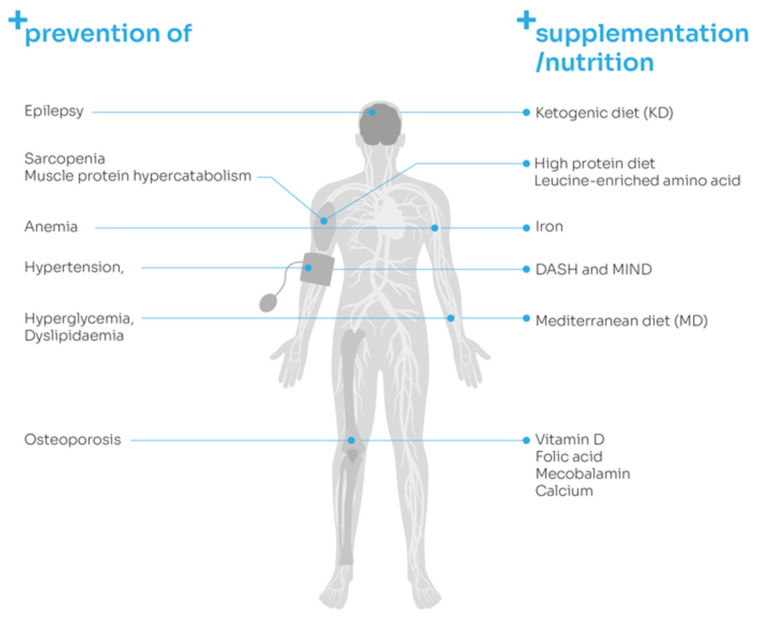
Potential preventive nutritional strategies in post-stroke patients.

Figure 2 summarizes preventive nutritional strategies in post-stroke patients presented in this review (Figure 2).

## 7. Agents from Marine Sources—A New Approach to Post-Stroke Neuroplasticity

Marine organisms are an excellent source of new chemicals, including a variety of primary and secondary metabolites with potent biological activity. Differences within species living in different marine environments may allow for compound variations and concentrations. Sea algae, whose metabolites have anti-inflammatory, antioxidant and antiamyloidogenic properties and affect cholesterol homeostasis, are worth noting in the context of their use as an adjuvant in the treatment of stroke. Thus, they have been shown to reduce neurotoxicity caused by glutamate, hydrogen peroxide and glucose or oxygen deficiency (Table 3) [90].

Fucoxanthin, contained in edible brown seaweed, decreased stroke volume, neurological deficits and the expression of proapoptotic proteins by activating the Nrf2/HO-1 pathway in the rat model of stroke [91]. In vitro studies have shown that fucoxanthin has an antioxidant effect by increasing the expression of superoxide dismutase and the production of glutathione, which was associated with the inhibition of H_2_O_2_-induced DNA damage [92]. Furthermore, fucoxanthin promoted cell survival through enhancement of BDNF bioavailability and activation of cAMP-dependent protein kinase (PKA)/cAMP response element-binding (CREB) pathway [98]. In animal models, fucoxanthin attenuated scopolamine-induced cognitive impairment, possibly by inhibiting acetylcholinesterase activity and modulating BDNF expression [94]. Moreover, it has been shown that this compound inhibits LPS-induced microglial neuroinflammation by reducing the secretion of inflammatory mediators—interleukin (IL) 1β, IL-6, tumor necrosis factor alpha (TNF-α), nitric oxide (NO) and prostaglandin E2 (PGE2). The anti-inflammatory activity of fucoxanthin is manifested by attenuating the mitogen-activated protein kinase (MAPK)/activating protein-1 (AP-1) and protein kinase B (Akt)/NF-κB pathways, as well as by lowering the expression of cyclooxygenase 2 (COX2) and inducible nitric oxide synthase (iNOS) [92,98].

Tramiprosate, found in red marine algae, decreased infarct volume in a dose-dependent manner in the rat stroke model. It was observed that tramiprosate at a dose of 50 mg/kg restored neurological deficits, and the time window was at least 6 h. A potential neuroprotective mechanism of these phytochemicals may be related to NMDA receptor (NMDAR) inhibition and nNOS translocation from the cytosol to the membrane without impact on total nNOS expression [95].

In addition, phlorotannins, polyphenols contained in brown algae, are popular foods in East Asian countries due to their strong antioxidant and anti-inflammatory properties. It has been demonstrated that *Ecklonia cava* polyphenols (ECP) dose-dependently (10 and 50 mg/kg) reduced brain edema and infarct volume and improved neurological dysfunction in the rat model of stroke. ECP inhibited neuronal apoptosis and promoted cell survival by inhibiting Ca^2+^-dependent neurotoxicity in a rat stroke model [96]. Similarly, ECP blocked the dose-dependent glutamate-mediated neuronal cytotoxicity (1–50 mg/kg). Moreover, ECP showed an antioxidant effect as evidenced by a reduction in mitochondrial Ca^2+^ overload and reactive oxygen species, increased mitochondrial membrane potential (ΔΨ_m_) and increased expression of heme oxygenase (HO-1) through Nrf2 nuclear translocation [97].

## 8. Conclusions

Nutritional interventions in post-stroke patients are a very important issue in patient care during the complex rehabilitation process, which includes functional training, occupational therapy, speech and cognitive therapy and physical medicine procedures. Post-stroke patients, especially the elderly, often suffer from nutrition-related diseases such as sarcopenia, osteoporosis, anemia and diabetes mellitus; therefore, there is a need to conduct nutritional interventions concomitant with rehabilitation. Future therapies, such as agents from marine sources, which are highly safe to consume, might be also included as potential neuroprotectants with antioxidative and anti-inflammatory properties. Therefore, the present narrative review highlights particular compounds that can be used in everyday clinical practice to improve patients’ responses to the high demand of a multitasking rehabilitation program. However, most of the presented clinical studies were conducted using small groups of participants, with clinical scales where the score of patient symptoms is somewhat subjective and not so precise. Moreover, there is a huge problem with the assessment of nutritional status due to a lack of valid markers of malnutrition. There is a need for future multicenter studies, with long-term observations, to create nutritional recommendations for post-stroke care and rehabilitation.

## Figures and Tables

**Table 1:** The correlation between nutritional status and functional recovery.

Study, Year, Reference	Study Design	Group 1	Group 2	Group 3	Outcome Measures	Main Findings
Kokura et al., 2016 [76]	Retrospective cohort study; *n* = 540	High GNRI < 92; *n* = 480	Low GNRI ≥ 92; *n* = 60	-	FIM	1 Patients with high nutritional-related risk at admission had lower FIM gain. 2 High nutritional-related risk may predict poor functional recovery.
Nishioka et al., 2016 [77]	cross-sectional study *n* = 178	MNA-SF NI	MNA-SF LI	MNA-SF GI	FIM	FIM efficiency was significantly higher in the GI group compared with the LI and NI groups. FIM gain was significantly lower in the NI group compared with LI and GI. The GI group had a significantly higher rate of home discharge.
Shimazu et al., 2021 [78]	single-left prospective cohort study; *n* = 454	low- frequency dietary prescription	high- frequency dietary prescription	-	FIM	Patients in the high-frequency group had lower FIM-motor scores at discharge but greater FIM-motor gains than low-frequency group.
Rabadi et al., 2008 [81]	Clinical trial, *n* = 116	intensive nutritional supplementation	standard nutritional supplementation	-	FIM, 2-minute and 6-minute timed walk tests	Intensive nutritional supplementation group improved more than standard nutritional supplementation group in motor function.

GNRI: Geriatric Nutritional Risk Index; FIM: Functional Independence Measure; MNA-SF: Mini Nutritional Assessment Short-Form; NI: no improvement; LI: lesser improvement; GI: greater improvement.

**Table 2:** The correlation between supplementation and functional recovery during rehabilitation.

Study, Year, Reference	Study Design	Group 1	Group 2	Outcome Measures	Main Findings
Yoshimura et al., 2019 [38]	RCT; *n* = 44	Supplement composition: 3 g of leucine 40% enriched essential amino acids and 9.7 g of carbohydrate	No supplementation	FIM, SMI, handgrip strength	↑ FIM score, significantly greater improvement in the IG than in the CG (*p* < 0.045). ↑ handgrip strength, significantly greater improvement in the IG (*p* < 0.01). ↑ SMI in the IG but not in the CG over time, significantly greater improvement in the intervention group.
Ikeda T, et al., 2020 [84]	RCT; *n* = 69	3.5 g of amino acids, 6.5 g of protein and 40 IU of vitamin D per 125 mL at breakfast	3.5 g of amino acids, 6.5 g of protein and 40 IU of vitamin D per 125 mL post-exercise	skeletal muscle mass, lower limb isometric strength, grip strength, TUGT, BBS, FIM	The effect of supplementation on muscle mass was similar in both groups. BCAA intake with breakfast and an exercise program was effective at improving physical performance and decreasing body fat mass. Ingestion of BCAAs with breakfast is effective for promoting rehabilitation of post-stroke patients.
Utkan Karasu 2021 et al., [88]	*n* = 76	50,000 IU of vitamin D weekly for 4–12 weeks	No supplementation	FAC, BRS lower extremity	↑ changes in FAC and BRS scores in group 1 (*p* = 0.005 and *p* = 0.018). ↑ changes in FAC and BRS scores in patients who were undergoing rehabilitation for the first time and/or in the first 3 months after stroke in group 1 than group 2 (*p* < 0.05). In patients > 3 months after stroke, vitamin D treatment did not affect FAC and BRS scores.
Gupta et al., 2016 [86]	RCT; *n* = 73	Vitamin D (600,000 IU single intramuscular injection and 60,000 IU once a month) + calcium (one gram per day) for 6 months	No supplementation, only usual care	Modified Rankin scale	Patients supplemented with vitamin D and calcium had better results in modified Rankin scale than control group receiving only usual care.
Sari et al., 2018 [85]	RCT; *n* = 132	300,000 IU vitamin D injection	saline intramuscular injection	BRS, FAS, MBI, BBS	The BBS results and MBI scores significantly differed between the two groups (higher scores in vitamin D group), but BRS and FAS test results did not significantly differ.
Momosaki et al., 2019 [89]	RCT; *n* = 100	Vitamin D (2000 IU per day) 8 weeks	placebo	Barthel index	The mean gain in the Barthel index score: 19.0 ± 14.8 in group 1 and 19.5 ± 13.1 in group 2 (*p* = 0.88). The Barthel index efficiency was 0.32 ± 0.31 in group 1 and 0.28 ± 0.21 in group 2 (*p* = 0.38). There was no significantly higher improvement in rehabilitation outcomes in the supplemented group.

↑: increase; RCT: randomized controlled trial; FIM: Functional Independence Measure; SMI: skeletal muscle mass index; IG: intervention group; CG: control group; BCAA: branched-chain amino acid; TUGT: timed up-and-go test; BBS: Berg balance scale; BRS: Brunnstrom recovery staging; FAS: functional ambulation scale; MBI: modified Barthel index; FAC: functional ambulation classification.

**Table 3:** Neuroprotective action of compounds from marine sources.

	Study, Year, Reference	Study Design	Supplementation	Outcome Measures	Main Findings
**Fucoxanthin (Fx)**	Hu et al., 2018 [91]	MCAO rat	Intragastrically administrated; 30, 60 and 90 mg/kg Fx; 1 h before MCAO induction	Infarct area; neurological function; brain water content of rats	Effect in dose-dependent manner Improvement of the neurologic deficit Decrease in the infarct volume Reduction in the level of apoptosis-associated proteins in brain tissues
		In vitro study OGD/R model (rat cortical neuron)	5, 10 and 20 μM Fx	Oxidative stress level; apoptosis level	Inhibition of increased caspase 3 expression Decrease in Bcl-2/Bax ratio Increase in SOD activity Decrease in MDA level Inhibition of OGD/R-induced apoptosis Decrease in ROS accumulation
	Pangestuti et al., 2013 [92]	In vitro study (amyloid-β42-induced BV2 microglia cells)	5, 10 and 50 μM Fx	Level of oxidative stress and inflammation	Inhibition of phosphorylation of MAPK pathway Inhibition of free radical-induced DNA oxidation Decrease in intracellular ROS production Increase in antioxidative enzymes activity
	Zhou et al., 2017 [93]	In vitro study (BV-2 cells)	5, 10 and 20 μM Fx	Anti-inflammatory, antioxidant and neuroprotective effect	Effect in dose-dependent manner Inhibition of proinflammatory mediators, both protein and mRNA expression: TNF-α, IL-6, PGE2, ROS, NO and COX, iNOS Inhibition of Akt/NFκB and MAPK/AP-1 pathways Promotion of BDNF production
	Lin et al., 2016 [94]	Institute of Cancer Research (ICR) mice	Six groups: (1) control (2) 3 mg/kg scopolamine (3) 3 mg/kg scopolamine + 50 mg/kg Fx (4) 3 mg/kg scopolamine + 100 mg/kg Fx (5) 3 mg/kg scopolamine + 200 mg/kg Fx (6) 3 mg/kg scopolamine + 3 mg/kg donepezil	Impact on scopolamine-induced cognitive impairments; impact on AChE activity; further examined if fucoxanthin could directly inhibit AChE in vitro	Improved cognitive impairments Decrease in AChE activity Decrease in choline acetyltransferase activity and BDNF expression Inhibition of AChE with an IC_50_ value of 81.2 μM
**Tramiprosate**	Wu et al., 2014 [95]	MCAO rats	50 mg/kg	Neuroprotective effect and impact on functional recovery	Effect in dose-dependent manner Reduction in the infarct volume Therapeutic window—6 h Improvement of neurological status Neuroprotective effect expressed by NMDAR Decrease in nNOS/PSD95 association Suppression of nNOS translocation to membrane
**Phlorotannin**	Kim et al., 2012 [96]	MCAO rats	Ecklonia cava polyphenols at 10 mg/kg and 50 mg/kg intraperitoneally administrated	Neuroprotective effect	Effect in dose-dependent manner Decrease in the extent of brain edema and infarct volume Inhibition of apoptosis Improvement of decreased neurological motor function
		In vitro study (differentiated neuroblastoma cell line SH-SY5Y)			Improvement of cell viability Decrease in H_2_O_2_-induced oxidative stress Inhibition of increased cytosolic calcium Reduction in calcium-induced neurotoxicity
	Cui et al., 2019 [97]	In vitro study (primary cortical neurons HT22 neurons)	100 µM, 24 h; 5 mM, 12 h	Neuroprotective effect	Effect in dose-dependent manner Enhancement of cell viability Recovery of neurons’ morphological deterioration Suppression of intracellular ROS level, disruption of mitochondrial membrane potential, overload of ROS and Ca^2+^ in mitochondria and ATP depletion Inhibition of oxidative stress Activation of Nrf2/HO-1 pathway
